# The Genotoxicity of Organic Extracts from Particulate Emissions Produced by Neat Gasoline (E0) and a Gasoline–Ethanol Blend (E15) in BEAS-2B Cells

**DOI:** 10.3390/jox14010001

**Published:** 2023-12-21

**Authors:** Helena Libalova, Tana Zavodna, Fatima Elzeinova, Hana Barosova, Tereza Cervena, Alena Milcova, Jolana Vankova, Foteini Paradeisi, Michal Vojtisek-Lom, Jitka Sikorova, Jan Topinka, Pavel Rossner

**Affiliations:** 1 Department of Nanotoxicology and Molecular Epidemiology, Institute of Experimental Medicine of the CAS, 14220 Prague, Czech Republic; helena.libalova@iem.cas.cz (H.L.); fatima.elzeinova@iem.cas.cz (F.E.); tereza.cervena@iem.cas.cz (T.C.); jolana.vankova@iem.cas.cz (J.V.); 2 Department of Genetic Toxicology and Epigenetics, Institute of Experimental Medicine of the CAS, 14220 Prague, Czech Republic; tana.zavodna@iem.cas.cz (T.Z.); hana.barosova@iem.cas.cz (H.B.); alena.milcova@iem.cas.cz (A.M.); fotiniparadissi@gmail.com (F.P.); jitka.sikorova@iem.cas.cz (J.S.); jan.topinka@iem.cas.cz (J.T.); 3 Centre of Vehicles for Sustainable Mobility, Faculty of Mechanical Engineering, Czech Technical University in Prague, 16000 Prague, Czech Republic; michal.vojtisek@tul.cz

**Keywords:** gasoline particulate emissions, organic PM extracts, alternative fuels, genotoxicity, polycyclic aromatic hydrocarbons, human pulmonary cell line

## Abstract

Emissions from modern gasoline engines represent an environmental and health risk. In this study, we aimed to compare the toxicity of organic compound mixtures extracted from particulate matter (PM extracts) produced by neat gasoline (E0) and a blend containing 15% ethanol (E15), which is offered as an alternative to non-renewable fossil fuels. Human lung BEAS-2B cells were exposed to PM extracts, and biomarkers of genotoxicity, such as DNA damage evaluated by comet assay, micronuclei formation, levels of phosphorylated histone H2AX, the expression of genes relevant to the DNA damage response, and exposure to polycyclic aromatic hydrocarbons (PAHs), were determined. Results showed that both PM extracts significantly increased the level of oxidized DNA lesions. The E0 extract exhibited a more pronounced effect, possibly due to the higher content of nitrated PAHs. Other endpoints were not substantially affected by any of the PM extracts. Gene expression analysis revealed mild but coordinated induction of genes related to DNA damage response, and a strong induction of PAH-inducible genes, indicating activation of the aryl hydrocarbon receptor (AhR). Our data suggest that the addition of ethanol into the gasoline diminished the oxidative DNA damage, but no effect on other genotoxicity biomarkers was observed. Activated AhR may play an important role in the toxicity of gasoline PM emissions.

## 1. Introduction

Motor vehicle exhaust emissions are of special concern due to their ubiquitous nature and high occurrence in urban areas, close to busy highways and major roads. They represent an important source of particulate matter (PM) in the atmospheric air, which has been associated with increased mortality and higher incidence of various diseases, including pulmonary and cardiovascular disorders as well as cancer [1].

Combustion-related aerosols consist of aggregated nuclei composed largely of elemental carbon with high concentrations of several toxic substances adsorbed on the surface, such as acid sulfates, soluble metals, and organic compounds, including polycyclic aromatic hydrocarbons (PAHs). Serious health risks are posed by particles with an aerodynamic diameter (d*ae*) below 2.5 µm (PM2.5) and particularly by an ultrafine fraction (d*ae* < 100 nm) because they can deposit deeper into the lungs, penetrate the bloodstream, and reach target organs [2]. 

PAHs are nonpolar aromatic compounds with two or more fused benzene rings that are generated by the incomplete combustion of organic material during industrial and other human-initiated activities, as well as natural processes. They represent extremely toxic components of PM emissions, with deleterious effects on human health. Several PAHs and their nitrated derivatives (nitro-PAHs) have been classified by the International Agency for Research in Cancer (IARC) as carcinogenic (group 1), probably carcinogenic (group 2A), and possibly carcinogenic (group 2B) to humans [3]. A metabolic activation is required to exert their mutagenic/carcinogenic effects. The oxidation of PAHs by P450 enzymes is the initial step in the activation process to produce polar highly reactive electrophilic intermediates (ultimate carcinogenic metabolites) that interact with cellular macromolecules, particularly nucleic acids and proteins. Genotoxicity is associated with the formation of stable or depurinating DNA adducts, or the production of ROS causing oxidative DNA damage [4]. A number of nitro-PAHs also exhibit carcinogenic and mutagenic properties. Some of them have even higher mutagenic potency than their parent PAHs due to their ability to act as a direct mutagen without metabolic activation [5]. 

Among the diverse PM produced by various combustion (engine emissions, biomass burning) and non-combustion sources (sea spray aerosols, ammonium sulfate, ammonium nitrate, secondary organic aerosols), diesel and gasoline exhaust PM was identified as the most toxic [6]. Recent findings showed that modern gasoline direct-injection (GDI) vehicles release a substantially higher amount of potentially toxic ultrafine PM compared to traditional port fuel injection vehicles and even compared to some diesel vehicles equipped with an after-treatment device, such as a diesel particle filter [7]. Diesel engine exhaust was classified by the International Agency for Cancer Research (IARC) as carcinogenic to humans (group 1) and gasoline engine exhaust as possibly carcinogenic to humans (group 2B) [8]. Therefore, detailed studies on genotoxicity and other toxic aspects of gasoline emissions are currently of special concern.

The use of alternative fuels derived from sources other than petroleum has become an issue of great importance, with an urgent need to reduce greenhouse gas emissions and reliance on the limited supply of fossil fuels. Ethanol, the most commonly used biofuel worldwide, is made from biomass, which is considered as a clean and renewable energy source. The addition of ethanol into gasoline has several advantages, e.g., an increased octane number, which improves the engine efficiency, and increased oxygen content, which promotes more complete combustion and therefore decreases the emissions of some pollutants such as carbon dioxide or hydrocarbons, including toxic PAHs and nitro-PAHs [9]. However, ethanol may conversely increase the production of highly toxic carbonyls such as acetaldehyde or formaldehyde [10]. It is thus not fully elucidated whether the toxicity of exhaust PM emissions from a gasoline–ethanol blend is always reduced in biological systems.

The objective of this study was to compare the toxicity of organic PM extracts originating from two different gasoline fuels: E0 (neat gasoline) and E15 (gasoline with an addition of 15% ethanol) in BEAS-2B cells, an in vitro model of the bronchial epithelium that has been used in numerous toxicological studies. We focused on the effect of the organic compound mixture extracted from PM, since this fraction is enriched with highly toxic PAHs and their derivatives, and tested the genotoxicity endpoints specific for PAHs.

## 2. Materials and Methods

### 2.1. PM Emission Collection and Processing

Detailed information about the utilized car, fuels, drive cycles, PM emission measurements, and sampling are given in a previous study [11]. Briefly, a typical European small family car (2013 Ford Focus, with a turbocharged gasoline direct-injection EcoBoost engine 92 kW@6000 rpm, certified to Euro 6) with a 6-speed manual transmission was chosen for all tests. Two fuels were used in this study: non-oxygenated gasoline with an octane number of 95 (E0) and gasoline with 15% ethanol (E15). The vehicle was operated on a 4-wheel chassis dynamometer according to the Common Artemis Driving Cycle, the version with a maximum speed of 130 km/h. The whole Artemis cycle was repeated four times to collect representative samples of exhaust particulate emissions. The exhaust was routed into a full-flow dilution tunnel with a constant volume sampler (CVS) operating at 10.8 m^3^/min, from which samples were taken. Diluted exhaust from the tunnel was sampled on 8 in × 10 in (203 × 254 mm) Teflon-coated glass fiber filters (Pall TX40HI20-WW, Pall Corporation, Port Washington, NY, USA) at a 67.8 m^3^/h sampling rate using a pair of modified EcoTech 3000 high-volume samplers (Ecotech Pty Ltd., Melbourne, Australia). Particle size distributions were measured online with a fast mobility particle sizer (EEPS, Model 3090, TSI, Shoreview, MN, USA).

### 2.2. Chemical Analysis of PM Extracts

As previously indicated [11], organic compounds were extracted with dichloromethane using an automated extraction apparatus, Behr EF (BEHR, Düsseldorf, Germany), for 4 h. Aliquot parts of the crude extract were re-dissolved in the required volume of acetonitrile for HPLC/DAD and LC/MS-MS. For further experiments, extracts were dissolved in DMSO to obtain stock solution in a concentration of 50 mg PM/mL. The method of external standardization was used for the quantification of unsubstituted PAHs (46 compounds), oxygenated PAHs (9 compounds), nitro-PAHs (4 compounds), and dinitro-PAHs (4 compounds). The accuracy and precision of the analytical methods were determined by analyzing the standard reference material (SRM) 1650b (diesel PM; National Institute of Standards and Technology, Gaithersburg, MD, USA).

### 2.3. Cell Culture, Exposure Conditions, and Cytotoxicity

Human bronchial epithelial cells BEAS-2B (ATCC, Manassas, VA, USA) were grown in basal medium (BEBM, Lonza, Basel, Switzerland) and supplemented with a standardized set of growth factors (BEGM™ BulleKit™, Lonza, Basel, Switzerland). Cultivation flasks and plates were coated with BEBM containing fibronectin, collagen, and bovine serum albumin. Cells were treated with PM extracts in several concentrations, depending on the method: five concentrations for cytotoxicity (0.1, 1, 25, 50, and 100 µg/mL), three concentrations for the comet assay, apoptosis, and micronucleus test (1, 25, and 50 µg /mL), and one concentration for gene expression analysis and determination of γH2AX (50 µg/mL). The control cells were incubated either with 0.5% DMSO (Sigma-Aldrich, USA) or with a medium. Cells were kept at 37 °C and 5% CO_2_. The cytotoxicity was assessed after 24 h exposure using the WST-1 colorimetric assay (Roche Diagnostics, Mannheim, Germany). Procedures were performed according to the manufacturer‘s recommendations. The absorbance was measured using an ELISA plate reader (Tecan Trading, Männedorf, Switzerland) at a wavelength of 440 nm. Parallel cultures without treatment served as a control. The final absorbance values were calculated as the difference between the readings obtained for the treated cells and the blank values. No tested PM extract in any dose exhibited cytotoxicity.

### 2.4. DNA Damage by Comet Assay

To measure the DNA strand breaks and oxidative DNA lesions, an alkaline version of the comet assay with and without restriction enzyme FPG, respectively, was carried used. Cells were seeded in 6-well plates and incubated overnight. After removing the media and washing the cells with PBS, the cells were treated with the tested compounds. Post-incubation, detachment of the cells was achieved using a 0.25% Trypsin-EDTA solution (Sigma Aldrich, St. Louis, MO, USA), followed by a cold PBS wash and dilution of the cell suspension to a density of 900,000 cells/mL. The comet assay was performed following the protocol described in Novotna et al. [12]. Images were captured with a CCD-13008 camera (VDS, Vosskühler, Germany) attached to a BX51 fluorescence microscope (Olympus, Tokyo, Japan). Lucia G 4.81 software (Laboratory Imaging, Prague, Czech Republic) was used to score the comets. A total of 100 randomly selected cells, both with and without the FPG enzyme treatment, were analyzed per sample. Oxidative DNA damage was calculated by subtraction of the median values of cells treated with FPG from those treated with the buffer. The findings were presented as the percentage of DNA in the tail (% tail DNA).

### 2.5. RNA Isolation and qRT-PCR Analysis

The expression of selected genes was evaluated using qRT-PCR. BEAS-2B cells treated for 24 h with tested PM extracts were lysed, and the total RNA was extracted using the NucleoSpin RNA II Isolation Kit (Macherey-Nagel, Düren, Germany) according to the manufacturer’s instructions. The concentration of RNA was quantified with a Nanodrop ND-1000 Spectrophotometer (Thermo Fisher Scientific, Waltham, MA, USA). Total RNA (500 ng) was transcribed into cDNA using the Transcriptor High Fidelity cDNA Synthesis Kit (Roche, Germany). TATAA Probe GrandMaster^®^ Mix (TATAA Biocenter AB, Goteborg, Sweden) and Taqman™ gene expression assays (Thermo Fisher Scientific, USA) were used for the relative quantification of the expression of the following genes: *CDKN1A* (Hs00355782_m1), *CDKN2A* (Hs00923894_m1), *XRCC2* (Hs03044154_m1), *DDB2* (Hs03044953_m1), *CYP1A1* (Hs00153120_m1), *SERPINB2* (Hs01010736_m1), *p63* (Hs00978340_m1), *BIK* (Hs00154189_m1), *GADD45A* (Hs00169255_m1), *GPX3* (Hs01078668_m1), *ALDH3A1* (Hs00964880_m1), and *DDIT3* (Hs01090850_m1). A CFX384 Real-Time System (Bio-Rad, Hercules, CA, USA) was used to perform qRT-PCR analysis. Ct-values were obtained using the Second Derivative Maximum Method in the CFX Manager^TM^ software version 3.1 (Bio-Rad). Expression levels of target genes were normalized to the reference genes *B2M* (Hs00187842_m1) and *ACTB* (Hs99999903_m1). Each tested PM-treated sample or control sample consisted of three biological replicates and two technical replicates.

### 2.6. Apoptosis

Cells were grown on 6-well plates overnight before the treatment with the tested substances. Cells were collected with Accutase following the exposure, resuspended in Annexin binding buffer (Becton Dickinson, San Jose, CA, USA), labelled with Annexin V, and conjugated with Alexa 647 (Thermo Fisher Scientific, USA) for 15 min at room temperature in the dark. Prior to analysis, Hoechst 33258 (Sigma Aldrich) was added to the cell suspension to detect necrotic cells. Fluorescence was analyzed by flow cytometry using a BD LSR II instrument (Becton Dickinson). A minimum of 10,000 events were collected for each sample. The percentage of live, apoptotic, and late apoptotic/necrotic cells was calculated with FlowJo (Tree Star, Inc., Ashland, OR, USA).

### 2.7. Micronuclei Analysis

The genotoxicity of the tested compounds was determined using the cytokinesis-block micronucleus assay performed in the 8-well Lab-Tek™ Chamber Slide System. The co-treatment version of the cytokinesis-block micronucleus assay, with simultaneous treatment with compounds and cytochalasin-B (Sigma Aldrich) (concentration 1 μg/mL) for 28 h, was performed. At the end of the cultivation, approximately 90% of the media was aspirated from the chambers, and the cells were treated with a hypotonic solution of KCl (0.075 M, Sigma Aldrich) for 3–5 min and fixed with a mixture of pre-chilled (2–8 °C) methanol (Merck KGaG, Darmstadt, Germany) and acetic acid (Penta, Prague, Czech Republic) (3:1). After 2 min, the fixation solution was aspirated, and 0.2 mL of chilled (2–8 °C) methanol was added in the last step of fixation. After a further 2 min, the methanol was completely aspirated, the chamber block was removed, and the slide was allowed to dry for at least 20 min. After fixation, the slides were dried and stained by 5% Giemsa (Merck). Visual scoring using an Olympus BX41 microscope was performed to analyze the binucleated cells (BNCs) at a final magnification of 1000×. A total of 3 × 500 BNCs per each tested compound and concentration were evaluated, and the cytokinesis-block proliferation index (CBPI) was calculated to control for cell division. The aberrant cells were recorded using a Canon EOS600D camera. The results were expressed as a percentage of BNC with MN (% ABB).

### 2.8. Analysis of γH2AX by Flow Cytometry

Cells were grown on 6-well plates overnight prior to treatment with the tested substances. Following 24 h exposure, the cell culture media was removed, and cells were washed twice with PBS and detached using Accutase (Sigma Aldrich). Collected cells were centrifuged (200× *g*, 4 °C, 5 min) and washed with ice-cold phosphate-buffered saline (PBS). Cells were then fixed by the addition of ice-cold 70% ethanol dropwise while vortexing. Fixed cells were incubated at 4 °C overnight and stored at −20 °C. Before the flow cytometry measurement, cells were pelleted by centrifugation (300× *g*, 4 °C, 10 min) and washed with ice-cold PBS to remove residual fixative. The washed cells were permeabilized in saponin-based permeabilization buffer (BD Perm/Wash™ buffer, BD Biosciences, San Jose, CA, USA) containing 1% bovine serum albumin (BSA) in PBS to minimize non-specific binding. After 15 min, cells were washed with ice-cold PBS. Cells were subsequently incubated with a primary antibody (Anti-phospho Histone H2A.X (Ser139) Antibody, clone JBW301, Alexa Fluor^®^ 647, Sigma Aldrich) diluted in 1% BSA for 30 min in darkness at 4 °C (antibody dilution 1:200). After incubation, cells were washed with PBS to remove excess antibody. Finally, the cells were incubated for 1 h with Propidium Iodide (PI) staining (PI with RNAse A 1:10 and Saponin-based permeabilization buffer solution containing 1% BSA). Cells were analyzed using a flow cytometer BD LSR II instrument (Becton Dickinson). A minimum of 10,000 events per sample were acquired. FlowJo software (Tree Star, Inc.) was used to analyze the data.

### 2.9. Statistical Analysis

Log-transformed relative changes in normalized gene levels (log2FC) were calculated using the 2^−ΔΔCt^ method [13]. Statistical significance of the changes between the exposed groups and the control group was determined by the ordinary one-way ANOVA followed by Dunnett’s post hoc test using GraphPad Prism 9.5.0. software. Statistical significance was defined as *p* < 0.05 (*) and *p* < 0.01 (**).

## 3. Results

### 3.1. Characteristics of PM and Chemical Analysis of the Organic Extracts

A characterization of PM emissions as well as sampling and chemical analysis of the PM extracts were described previously [11]. Both fuels, E0 and E15, produced a comparable amount of PM emissions (1.70 vs. 1.76 mg/km for E0 and E15, respectively). The concentration of unsubstituted PAHs, including carcinogenic (benz[*a*]anthracene, benzo[*a*]pyrene, benzo[*b*]fluoranthene, benzo[*k*]fluoranthene, chrysene, dibenz[*a*,*h*]anthracene and indeno [1,2,3-*c*,*d*]pyrene) as well as other priority PAHs (anthracene, fluoranthene, phenanthrene, pyrene, benzo[*g*,*h*,*i*]perylene, etc.) was determined. We further measured the concentration of PAH derivatives such as oxygenated PAHs (9H-fluoren-9-one, anthracene-9,10-dione, 7H-benz[de]anthracene-7one, 1,8-naphthalic anhydride, etc.), nitrated PAHs (1-, 2-, 4-nitropyrene and 3-nitrofluoranthene) and dinitrated PAHs (1,3-, 1,6- and 1,8-dinitropyrene). The E0 extract contained a slightly higher sum of carcinogenic PAHs, but the sum of all unsubstituted PAHs was higher in the E15 extract due to the abundance of PAHs with lower molecular weight, such as phenanthrene, anthracene, fluoranthene, and pyrene. The sum of oxygenated PAHs was comparable in both extracts, while the sum of nitrated PAHs was substantially higher in the E0 extract. Dinitrated PAHs were not detected in any of the tested extracts. A summary of the results is presented in Table 1.

### 3.2. Determination of DNA Strand Breaks (DNA-SBs) and Oxidative DNA Damage

**Figure 1:**
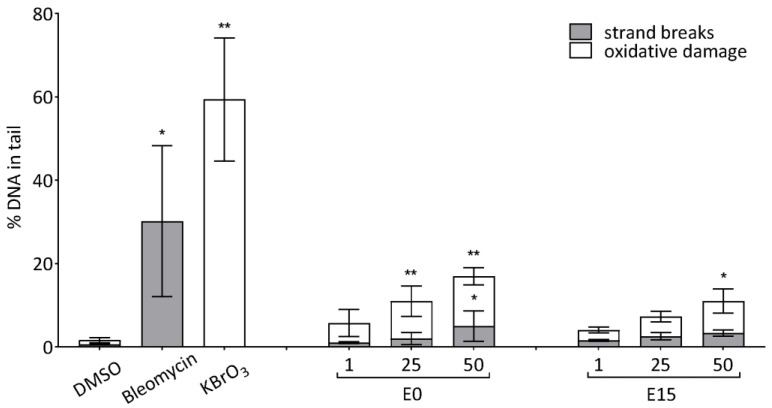
The effect of E0 and E15 extracts on the induction of DNA-SBs and oxidative DNA damage in BEAS-2B cells. DNA damage was determined by the comet assay following the incubation of cells with three doses of each extract (1, 25, and 50 µg/mL) for 24 h. Bleomycin (5 µM) and KBrO_3_ (1 mM)_,_ positive controls for strand breaks and oxidative damage, respectively, were incubated in cells for 1 h. Bars (% tail DNA) represent the median value ± S.D. of three independent experiments. Statistical analysis was performed using ANOVA. Symbols * and ** indicate a statistically significant difference compared to the respective control (* *p* < 0.05, ** *p* < 0.01).

BEAS-2B cells were exposed for 24 h to three non-cytotoxic doses of E0 and E15 extracts (1, 25, and 50 µg/mL), and DNA-SBs were determined by the alkaline version of the comet assay. To estimate the oxidative DNA damage, modification with DNA repair enzyme formamidopyrimidine DNA glycosylase (FPG) was used. Both PM extracts promoted the generation of DNA-SBs and oxidative damage in a dose-dependent manner (Figure 1). Whereas two higher doses of E0 (25 and 50 µg/mL) induced a significant increase of oxidized DNA lesions compared to the DMSO control (9 and 12% of DNA in tail, respectively, *p* < 0.01), the E15 extract was generally less effective: a significant impact was observed for the dose 50 µg/mL only (7.7% of DNA in tail, *p* < 0.05). DNA-SBs were also increased by both PM extracts, but only the highest dose of E0 induced a significant change (5.0%, *p* < 0.05).

### 3.3. Detection of Phosphorylated Histone H2AX

**Figure 2:**
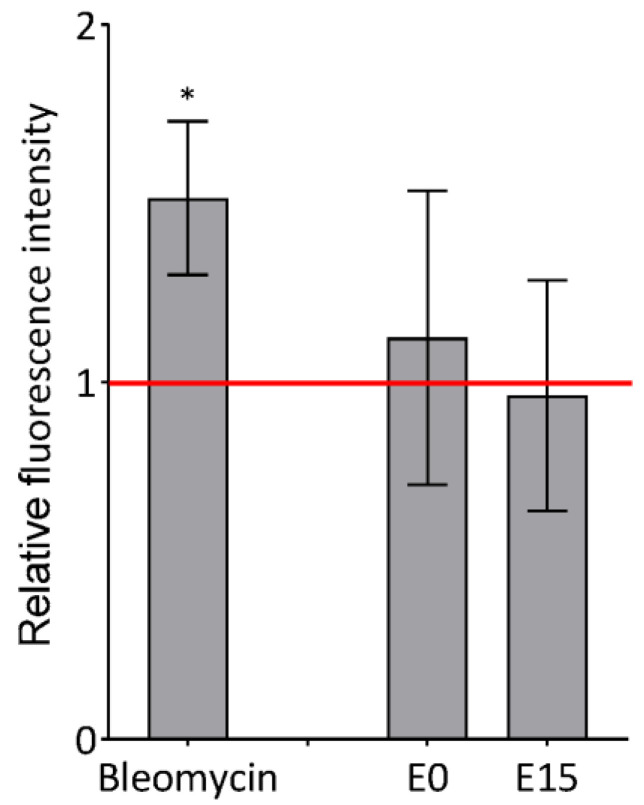
The effect of the 50 µg/mL dose of E0 and E15 extracts on the induction of DSB in BEAS-2B after 24 h exposure. One-hour incubation of cells with 5 µM bleomycin was used as positive control. Data are expressed as the mean of fluorescence intensity relative to the DMSO control from three independent experiments ± S.D. Statistical analysis was performed using ANOVA. The symbol * indicates a statistically significant difference compared to the respective control (* *p* < 0.05).

The level of phosphorylated histone H2AX (γH2AX), an established marker of DNA damage and repair that is also used for the assessment of double strand breaks (DSBs), was analyzed by flow cytometry in BEAS-2B incubated with E0 and E15 extracts with the dose 50 μg/mL for 24 h. As presented in Figure 2, none of the tested PM extracts showed significant induction of γH2AX fluorescence, indicating the absence of DSBs. The positive assay control (bleomycin) significantly increased the level of γH2AX (1.5-fold induction, *p* < 0.05).

### 3.4. Chromosomal Damage

**Figure 3:**
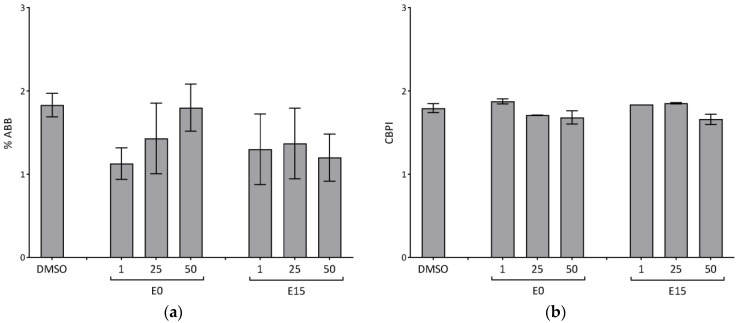
Micronuclei formation in BEAS-2B. Cells were exposed to three concentrations of E0 and E15 extracts (1, 25, and 50 µg/mL) for 28 h and analyzed for (**a**) aberrant binucleated cells with micronuclei (% ABB) and (**b**) CBPI. Data shown are expressed as the means from two independent experiments ± S.D. Statistical analysis was performed using ANOVA.

An increased frequency of micronuclei (MN) is considered a sign of severe DNA damage, leading to chromosomal instability. The cytokinesis block proliferation index (CBPI) is used as a parameter of cytotoxicity. The effect of PM extracts on the MN formation and CBPI in BEAS-2B cells versus the corresponding control cultures is shown in Figure 3. Basically, none of the extracts increased the frequency of MN in BEAS-2B cells after 28 h incubation; instead we observed a slight decrease of MN frequency with the decreasing dose of the E10 extract, and a constant decrease after the treatment with all doses of the E15 extract. However, the changes were not statistically significant. The CBPI index of BEAS-2B cells following the incubation with the highest doses of 50 μg/mL of both extracts showed a slightly decreasing tendency compared to the control, indicating possible cytotoxicity or cell cycle delay caused by the tested dose (Figure 3).

### 3.5. Apoptosis

**Figure 4:**
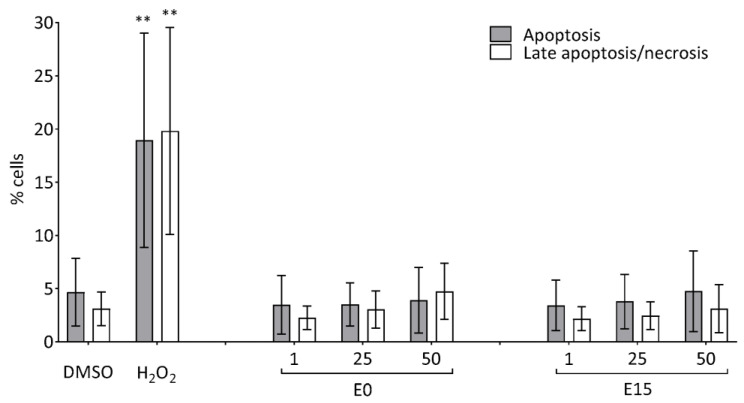
Apoptosis measured by flow cytometry in BEAS-2B cells after 24 h exposure to 1, 25, and 50 µg/mL of E0 and E15 extracts. Incubation of cells with 400 µM H_2_O_2_ for 15 h was used as a positive control. Results are presented as the percentage of apoptotic and late apoptotic/necrotic cells. Bars (% cells) represent the mean ± S.D. of three independent experiments. Statistical analysis was performed using ANOVA. The symbol ** indicates a statistically significant difference compared to the respective control (** *p* < 0.01).

The induction of apoptosis in cells following 24 h exposure to three doses of both PM extracts (1, 25, and 50 µg/mL) was evaluated. The percentage of apoptotic and necrotic cells was comparable in all samples, i.e., incubated with any of the PM extracts for 24 h or in control cells, excepting a subtle increase of necrotic cells induced by 50 µg/mL of E0 extract. Changes were not significant, and neither apoptotic nor necrotic cells exceeded 5% (Figure 4).

### 3.6. Gene Expression Changes

**Figure 5:**
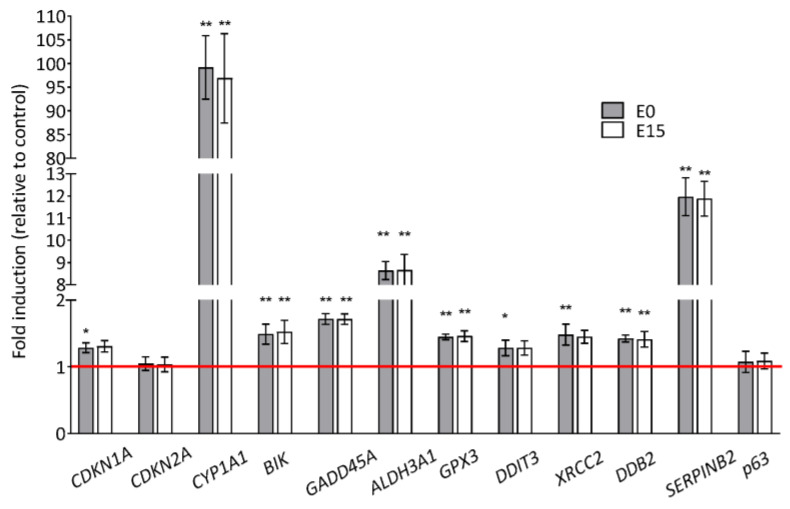
Gene expression changes following 24 h exposure to a concentration of 50 µg/mL of E0 and E15 extracts detected by qRT-PCR. Fold change (FC) was obtained by normalization to control samples (2^−ΔΔ^*^C^*^t^ method). Each FC represents a mean value of measurements in three independent samples; two technical replicates were also included. Statistical analysis was performed using ANOVA. Symbols * and ** indicate a statistically significant difference compared to the respective control (* *p* < 0.05 and ** *p* < 0.01).

To investigate the effects of PM extracts on gene expression changes, we analyzed the mRNA levels of 12 genes involved in the DNA damage response and activation of checkpoints (*GADD45A*, *DDIT3*, *CDKN1A*, *CDKN2A*, *p63*), oxidative stress (*GPX3*, *ALDH3A1*), DNA repair (*XRCC2*, *DDB2*), apoptosis (*BIK*), and metabolism of PAHs (*CYP1A1*, *SERPINB2*) using quantitative real-time PCR (qRT-PCR). The genes were previously identified by microarrays as significantly deregulated in response to E0 and E15 extract exposure [11]. As shown in Figure 5, the highest increase of gene expression, an almost 100-fold change (FC) compared to the control, was observed for *CYP1A1* followed by *SERPINB2* (approx. 12 FC, *p* < 0.01) and *ALDH3A1* (approx. 9 FC, *p* < 0.01) in the cells treated by both PM extracts. A less pronounced but statistically significant increase of expression was detected in other genes, such as *BIK*, *GADD45A*, *GPX3*, *XRCC2*, and *DDB2*, following incubation with both PM extracts (approx. 1.5 FC, *p* < 0.01). The expression of *CDKN1A* and *DDIT3* was significantly increased after the E0 extract treatment only (approx. 1.5 FC, *p* < 0.05), and the expression of two genes, *CDKN2A* and *p63*, was not affected by any of the extracts. No significant difference in expression levels of the selected genes was observed between the E0 and E15 extracts.

## 4. Discussion

In this study, we compared the toxicity of organic extracts from PM produced by the combustion of neat fossil gasoline (E0) and an alternative fuel containing 15% ethanol (E15) in BEAS-2B cells. Our intention was to evaluate the potential impact of the addition of ethanol on the genotoxic potential of PM extracts in human bronchial epithelial cells (BEAS-2B) and to relate the cellular response to the chemical composition of the extracts. We focused on the effect of carcinogenic PAHs, as they substantially contribute to the toxicity of PM. When inhaled, PM2.5 are deposited mainly in the pulmonary region; therefore, the bronchial epithelium represents a target organ of PAH exposure. BEAS-2B cells have been established as a metabolically competent model of bronchial epithelial cells and are widely used to study the genotoxic and carcinogenic properties of various substances.

In our previous study, it was demonstrated that both E0 and E15 extracts were enriched with a substantial amount of PAHs and their derivatives [11]. Whereas no significant difference in unsubstituted PAHs was observed between the extracts, a markedly higher quantity of nitro-PAHs, particularly 1-nitropyrene, was detected in the E0 extract. Although nitro-PAHs accounted for only a small part of the total PAH mass fraction in environmental mixtures, they are highly toxic, and even if presented in small amounts, they significantly contribute to the total genotoxicity [14]. Previously, diesel engine emissions have been considered as the dominant source of nitro-PAHs, and 1-nitropyrene has been used as a marker of diesel emissions [15]. However, recent studies showed that GDI vehicles also emit a high concentration of nitro-PAHs, even higher than diesel engines equipped with a particle filter [16,17]. The decrease of nitro-PAHs in E15 is in agreement with another study demonstrating a substantial reduction of PAHs and nitro-PAHs in emissions from GDI vehicles fueled with a gasoline–ethanol blend [9]. On the other hand, it should be noted that PAH emissions depend on various parameters, including fuel type, engine technology, conditions (warm or cold engine during starting, steady-state or transient operation) or after-treatment [9].

The comet assay represents an acknowledged tool for the evaluation of genotoxic properties. We applied an alkaline version that can reveal both single and double DNA-SBs, alkali labile sites, DNA cross-linking, and incomplete excision repair sites [18]. Modification of the comet assay with inclusion of FPG or endonuclease III enables recognition of the oxidized DNA bases. After 24 h, both the E0 and E15 extracts increased the level of DNA-SBs in a dose-dependent manner, although these changes were more pronounced and significant for the highest dose of E0 extract. Together with the detection of γH2AX indicating no presence of DSBs, we thus inferred that DNA-SBs were mostly single-stranded. E0 further induced a substantial amount of oxidized DNA lesions, while E15 was less effective. In a study by Novotna et al. [12], DNA-SB levels as an indicator of genotoxic potency were measured by the comet assay in A549 cells incubated with PM extracts from conventional fossil diesel (B0) and neat biodiesel (B100) emissions. It was found that despite higher levels of DNA-SB and an enormous increase in oxidative DNA damage induced by B100 extract after 4 h incubation, B0 was more genotoxic than B100 after 24 h incubation. This was related to an intensive and rapid DNA repair after 4 h, less severe DNA lesions induced by B100, and higher content of PAHs in B0 extract. In our study, despite using extracts from PM generated by gasoline engines, which generally produce much less particulate black carbon and the associated organic matter than diesel engines, we observed a similar trend when compared to the neat gasoline and the gasoline–ethanol blend. Our data suggest that both extracts induced persistent DNA lesions, but the E0 extract exhibited higher genotoxic potency and increased oxidative DNA damage. Recently, we tested the genotoxic potential of two model carcinogenic PAHs, benzo[*a*]pyrene (B[*a*]P) and 1-nitropyrene (1-NP), in BEAS-2B cells using the comet assay. Our results demonstrated that both PAHs considerably increased the level of DNA-SB and particularly oxidized lesions (up to 30% of DNA in the tail). Also, 1-NP showed higher oxidative potential than B[*a*]P (data not published). The high pro-oxidant effect of 1-NP was also confirmed in A549 cells [19]. Given the fact that both extracts contained similar content of unsubstituted PAHs, but 1-NP was substantially higher in the E0 extract, we thus speculate that 1-NP may be responsible for such a difference. Our results, indicating a lower genotoxic potency of the gasoline–ethanol blend, are consistent with other relevant studies comparing the toxicity of gasoline and biogasoline PM emissions. Agarwal et al. [20] reported that the cytotoxicity and mutagenicity of biogasoline PM were lower than gasoline PM, and this was correlated with a lower content of PAHs. In a study by Yang et al. [16], the intrinsic oxidative potential was lower for higher ethanol blend E78 than for lower blends containing a higher proportion of aromatic compounds, PM, and gaseous toxins.

We further performed measurement of the mRNA levels of specific genes involved in processes related to DNA damage and oxidative stress response, DNA repair, and metabolism of PAHs that were previously identified by microarray analysis as significantly deregulated [11]. A massive increase of *CYP1A1* expression was detected following exposure to both PM extracts. CYP1A1 is a key enzyme involved in the metabolic transformation of PAHs to epoxide intermediates, which are converted into diol-epoxides, ultimate carcinogens that form covalent DNA adducts. It further participates in the formation of radical cations that are converted into quinones. These metabolites undergo redox cycling and generate ROS that react with guanine and cause oxidative DNA damage [4]. *CYP1A1* expression is regulated in a substrate-inducible manner by aryl hydrocarbon receptor (AhR). The activation of AhR is a major toxic mode of action of many PAHs as well as their complex mixtures [21]. The AhR-mediated toxicity includes many effects, such as cross-talk with signaling pathways and disruption of cell cycle progression and apoptosis, that further contribute to their carcinogenic properties [22]. In our previous study, we demonstrated that the genotoxicity of a complex mixture containing PAHs was inhibited by other mixture components in comparison to single PAHs, but AhR activity remained unchanged or rather was enhanced [23]. Accordingly, several high-molecular PAHs (MW302) have been shown to exhibit high AhR-mediated activities, while the genotoxicity was limited [24]. Therefore, the effect of PM extracts observed in our study may not necessarily reflect their overall toxicity and carcinogenicity, as we focused on genotoxicity only.

Another gene participating in the metabolism of PAHs and significantly upregulated by both extracts was *ALDH3A1*. ROS generated during the oxidation of PAH metabolites are sources of oxidative damage of DNA, proteins, and lipids [25]. ALDH3A1 is responsible for the detoxification of reactive aldehydes, which are derived from lipid peroxidation [26]. Similarly to *CYP1A1*, *ALDH3A1* also contains a xenobiotic responsive element in a gene promoter, and its transcription is therefore regulated by activated AhR. Various PAH-complex mixtures, such as cigarette smoke extract [27] or extract from ambient air respirable particles PM2.5 [23], have been shown to activate *ALDH3A1*. Expression of another marker of oxidative stress, *GPX3*, was also enhanced to a lesser but significant extent by both extracts. Taken together, this indicates the pro-oxidant potential of organic compounds extracted from gasoline exhaust PM. The pro-oxidant properties of organic extract from ambient respirable particles (PM2.5) containing PAHs in BEAS-2B cells were also demonstrated in a recent study [28].

SERPINB2 is well described as an inhibitor of urokinase plasminogen activator (uPA) and tissue plasminogen activator (tPA) but is also involved in multiple processes, such as signal transduction, inhibition of apoptosis and necrosis, regulation of monocyte and keratinocyte proliferation and differentiation, cyto-protection, and modulation of immune responses [29]. It has been demonstrated that *SERPINB2* is a direct downstream target of p53, activated via the DNA damage response pathway [30], and plays a role in UV-induced nucleotide excision repair (NER) [29]. In another study [31], the authors found that the expression of the *SERPINB2* gene is regulated by activated AhR. We thus consider *SERPINB2* as another relevant marker of PAH exposure and toxicity related to activated AhR as well as an indicator of genotoxic stress.

DNA damage response (DDR) is one of the key stress response pathways, comprising DNA repair, cell-cycle checkpoint control, and DNA damage-induced senescence and apoptosis, which protect genomic integrity and suppress tumorigenesis. The tumor suppressor p53 has a critical role in DDR; it functions as a transcription factor and activates multiple cellular processes, including cell cycle arrest, apoptosis, and senescence [32]. Depending on the level and type of DNA damage, p53 selectively modulates the transcription of distinct batteries of target genes, thus promoting cell survival or cell death [33]. A mild but significant and coordinated increase of several p53 targets was detected in our study, indicating the activation of p53 signaling. GADD45A has a growth suppression function, while BIK and DDIT3 are implicated in apoptosis induced by genotoxic stress. CDKN1A is a critical effector of p53 that mediates cell cycle arrest and can also inhibit apoptosis. DDB2 is involved in NER, a primary defense against bulky PAH-DNA adducts [34]. We further observed an increased mRNA level of *XRCC2*, gene coding the key protein participating in homologous recombination, a repair mechanism that removes DSBs and inter-strand DNA crosslinks. HR also enhances DNA damage tolerance by mechanisms that circumvent the lesion, postpone their repair, and enable the completion of DNA replication in time to prevent a replication fork breakage, which leads to genomic instability [35]. Alternatively, expression of *p63*, gene coding the transcription factor belonging to the p53 family, which has an ability to compensate for p53 loss, and *CDKN2A*, gene coding an inhibitor of cell cycle and regulator of p53 activation, was not affected. This may further support the hypothesis that although p53 signaling was activated, the DDR did not lead to cell death, and DNA lesions were either effectively repaired or tolerated.

Despite increased levels of DNA lesions and the enhanced expression of p53-dependent genes by both extracts, no effect on apoptosis and the formation of micronuclei was observed. This might be related to the absence of the most lethal DSB, as indicated by the absence of histone H2AX phosphorylation. As mentioned above, we observed a substantially increased level of oxidized DNA lesions in our study. It has been shown that p53 functions as a coordinator of the oxidative stress response; in the case of low levels of oxidative stress, p53 contributes to the elimination of ROS and promotes cell survival, while at a high level, p53 exhibits rather pro-oxidant activities that further increase the levels of stress and lead to cell death. An overexpression of antioxidant genes, such as *GPX* and *ALDH*, which was similarly observed in our study, was associated with low stress and p53-mediated antioxidant activity [36]. This may indicate that a low or moderate level of oxidative stress led to cellular recovery rather than cell death.

## 5. Conclusions

In summary, we found that the addition of ethanol into the neat gasoline changed the chemical composition of PM emissions from the GDI vehicle and affected the genotoxicity of the organic compound mixture in BEAS-2B cells. E0 induced a higher level of DNA-SB and particularly oxidized DNA lesions in comparison to E15, and this effect corresponded to a higher content of nitro-PAHs found in E0. Despite the enhanced expression of selected genes related to p53 signaling, oxidative stress, and metabolism of PAHs, neither PM extract caused phosphorylation of H2AX, indicating the absence of DSB, the formation of micronuclei, or induced apoptosis, possibly due to the p53-mediated antioxidant activity, resulting in a pro-survival effect. We further detected an increased expression of genes indicating the activation of AhR, which may play an important role in the toxicity of gasoline PM extracts. These results may contribute to general knowledge on the impact of the ethanol additive on human health.

## 6. Study Limitations

In our study, we determined the genotoxicity of the organic fraction extracted from particles and aimed to correlate the results with the content of PAHs. However, the effect of the whole PM was not taken into account. The exposure of cells to the whole PM provides an undisputable advantage due to the complexity of the toxic response; however, this approach has some shortcomings, such as the problematic obtainment of intact particles from filters, inaccurate dosage, the aggregation of PM, and the impossibility of distinguishing the effect of core particles and compounds bound on the PM surface. To credibly simulate realistic conditions, several studies recently established a new approach involving the exposure of complete PM emissions from gasoline and various gasoline–ethanol blends on cells or cell cocultures grown on the air–liquid interface. Despite this, our study emphasizes the important role of PAHs in the genotoxicity of gasoline emissions and contributes to the complex knowledge on their adverse effect on human health.

## Figures and Tables

**Table 1:** Emission characteristics of PM generated from E0 and E15 fuels and chemical analysis of PAH compounds and their oxygenated, nitrated, and dinitrated derivatives in PM extracts.

Emission Characteristic	E0	E15
PM mass (mg/km)	1.70	1.76
Sum of PAHs (ng/mg PM)	959	999
Sum of carcinogenic PAHs ^1^ (ng/mg PM)	257.1	244.8
Sum of oxygenated PAHs (ng/mg PM)	207.5	215.7
Sum of nitrated PAHs (pg/mg PM)	231	142
Sum of dinitrated PAHs (pg/mg PM)	0	0

^1^ Carcinogenic according to IARC.

## Data Availability

The data presented in this study are available on request from Helena Libalova (helena.libalova@iem.cas.cz).

## Abbreviations

1-NP, 1-nitropyrene; AhR, Aryl hydrocarbon receptor; B[*a*]P, Benzo[*a*]pyrene; BNC, Binucleated cells; BSA, Bovine serum albumin; CBPI, Cytokinesis block proliferation index; CVS, Constant volume sampler; DDR, DNA damage response; DMSO, Dimethyl sulphoxide; DNA-SB, DNA strand breaks; DSB, Double strand breaks; E0, Neat gasoline; E15, Gasoline with an addition of 15% ethanol; E20, Gasoline with an addition of 20% ethanol; FC, Fold change; FPG, Formamidopyrimidine DNA glycosylase; GDI, Gasoline direct injection; HPLC/DAD, High-performance liquid chromatography with diode-array detection; IARC, International agency for research on cancer; LC/MS-MS, Liquid chromatography with tandem mass spectrometry; MN, Micronuclei; NER, Nucleotide excision repair; PAH, Polycyclic aromatic hydrocarbons; PM, Particulate matter; qRT-PCR, Quantitative real-time polymerase chain reaction; ROS, Reactive oxygen species; RT, Room temperature; tPA, Tissue plasminogen activator; uPA, Urokinase plasminogen activator; γH2AX, Phosphorylated histone H2AX.
